# Tactile Cross-Modal Acceleration Effects on Auditory Steady-State Response

**DOI:** 10.3389/fnint.2019.00072

**Published:** 2019-12-17

**Authors:** Shunsuke Sugiyama, Tomoaki Kinukawa, Nobuyuki Takeuchi, Makoto Nishihara, Toshiki Shioiri, Koji Inui

**Affiliations:** ^1^Department of Psychiatry and Psychotherapy, Gifu University Graduate School of Medicine, Gifu, Japan; ^2^Department of Anesthesiology, Nagoya University Graduate School of Medicine, Nagoya, Japan; ^3^Depatment of Psychiatry, Aichi Medical University, Nagakute, Japan; ^4^Multidisciplinary Pain Center, Aichi Medical University, Nagakute, Japan; ^5^Departmernt of Functioning and Disability, Institute for Developmental Research, Kasugai, Japan

**Keywords:** ASSR, audio-tactile, cross-modal, latency, MEG

## Abstract

In the sensory cortex, cross-modal interaction occurs during the early cortical stages of processing; however, its effect on the speed of neuronal activity remains unclear. In this study, we used magnetoencephalography (MEG) to investigate whether tactile stimulation influences auditory steady-state responses (ASSRs). To this end, a 0.5-ms electrical pulse was randomly presented to the dorsum of the left or right hand of 12 healthy volunteers at 700 ms while a train of 25-ms pure tones were applied to the left or right side at 75 dB for 1,200 ms. Peak latencies of 40-Hz ASSR were measured. Our results indicated that tactile stimulation significantly shortened subsequent ASSR latency. This cross-modal effect was observed from approximately 50 ms to 125 ms after the onset of tactile stimulation. The somatosensory information that appeared to converge on the auditory system may have arisen during the early processing stages, with the reduced ASSR latency indicating that a new sensory event from the cross-modal inputs served to increase the speed of ongoing sensory processing. Collectively, our findings indicate that ASSR latency changes are a sensitive index of accelerated processing.

## Introduction

Animals must simultaneously process various multisensory information, including that related to visual, auditory and tactile stimuli. Recent studies showed that these multisensory interactions occur during the early cortical stages of processing (Schroeder and Foxe, 2005; Macaluso, 2006; Driver and Noesselt, 2008; Alais et al., 2010) in brain regions that were previously considered unisensory (Ghazanfar and Schroeder, 2006). Indeed, studies using functional magnetic resonance imaging (fMRI; Calvert et al., 1997; Foxe et al., 2002; van Atteveldt et al., 2004; Pekkola et al., 2005), event-related potentials (ERPs; Giard and Peronnet, 1999; Foxe et al., 2000; Molholm et al., 2002; Besle et al., 2004; van Wassenhove et al., 2005) and magnetoencephalography (MEG; Lütkenhöner et al., 2002; Gobbelé et al., 2003) reported that visual and somatosensory interactions occur in the human auditory cortex. Furthermore, studies using macaques established the presence of audiovisual and audio-tactile convergence in the subregions of the auditory cortex (Schroeder et al., 2001; Schroeder and Foxe, 2002), as well as the presence of neurons in the primary auditory cortex that respond to both auditory and somatosensory stimuli (Fu et al., 2003).

The results of these studies indicated that the convergence of sensory information from different modalities occurs during the early stages of sensory processing. However, whether signals from other sensory systems modulate the processing speed of the human auditory cortex remains largely unknown. Studies focusing on neuronal oscillations reported that neurophysiological mechanisms underlie early multisensory interactions (Lakatos et al., 2007, 2008, 2009; Kayser et al., 2008; Romei et al., 2012; Mercier et al., 2015). Using neural oscillations, some of these studies reported an acceleration of the cross-modal interaction. Indeed, Mercier et al. (2015) recorded electrocorticograms and reaction times in patients who are epileptic when auditory, visual, or audiovisual stimuli were simultaneously presented, and they found that higher synchronization in the auditory area results in faster response times. These findings indicate an important role for cross-modal interactions in the multisensory facilitation of reaction times. In addition, a recent source density study using monkeys demonstrated the correlation between the phase of delta oscillation and reaction time (Lakatos et al., 2008).

In this study, we recorded auditory steady-state responses (ASSRs) in order to investigate the acceleration effects of tactile inputs. Steady-state responses (SSRs) are believed to be electrophysiological responses that are driven by a train of stimuli delivered at a markedly high rate, with ASSRs reaching a maximum amplitude of approximately 40 Hz (Galambos et al., 1981; Ross et al., 2000). Previous studies using MEG (Ross, 2008) and positron-emission tomography (Pastor et al., 2002) reported that ASSRs originate in the primary auditory cortical areas or subcortical areas (Herdman et al., 2002). Phase resetting is the term used for the impact of a salient sensory stimulus on an SSR, with this process inducing the modulation of the SSR amplitude and phase. Rohrbaugh et al. (1989, 1990a,b) investigated the impact of a foreground auditory or visual stimulus on a 40-Hz ASSR evoked by a background rhythmic probe stimulus, and they found a reduction of both the amplitude and latency of the resulting ASSRs. In addition, Makeig and Galambos (1989) reported that similar phase resetting occurs in 40-Hz ASSRs with sudden variations in the frequency or intensity of the stimuli train. In a study using an auditory oddball paradigm, button pressing in response to a rare stimulus also caused phase resetting in 40-Hz ASSRs (Rockstroh et al., 1996). Furthermore, Ross et al comprehensively examined phase resetting and established that ASSRs are modulated by various factors, including stimulus onset (Ross et al., 2002), variations in the periodicity of the sound stimulus (Ross and Pantev, 2004), and the presence of an interfering stimulus (Ross et al., 2005a). Our recent study showed that phase shifts of ASSR depended on the magnitude of sound-pressure change (Motomura et al., 2019). Using an oddball paradigm, we reported that ASSR latency can be shortened without changes in peripheral inputs. This novel finding indicated that sensory memory and comparison processes could occur in brain areas higher than the primary cortex in terms of acceleration effects, with faster processing in ASSRs contributing to shorter reaction times (Sugiyama et al., 2019).

In this study, we aimed to investigate whether phase resetting of ASSRs, particularly the temporal aspect, is affected by tactile stimulation. While Rohrbaugh et al. (1990a) demonstrated a possible influence of a visual flash on ASSRs, Makeig and Galambos (1989) demonstrated that the phase shifts of auditory and visual SSRs were only observed with stimuli of the same modality. Furthermore, to our knowledge, no study has investigated the impact of tactile stimulation on ASSRs, which are considered superior to the middle latency components of auditory evoked magnetic fields for observing subtle changes in processing timing (Sugiyama et al., 2019). In addition, MEG methods can record ASSRs in the millisecond range, rendering this technique useful for assessing the impact of acceleration on ASSRs. Therefore, we hypothesized that tactile stimulation may decrease ASSR latency because of the accelerative nature of cross-modal interactions (Rowland et al., 2007).

## Materials and Methods

This study was approved in advance by the Ethics Committee of the National Institute for Physiological Sciences, Okazaki, Japan, and it was conducted in accordance with the Declaration of Helsinki. Written informed consent was obtained from all subjects. The study was conducted on 12 healthy volunteers (three females and nine males) aged 22–38 (mean, 29.0) years. None of the subjects presented with any history of mental or neurological disorders or substance abuse in the last 2 years, and they were free of medication at testing. In addition, participants had a hearing threshold lower than 30 dB at 1,000 Hz as assessed by an audiometer (AA-71, Rion, Tokyo, Japan).

### Auditory and Tactile Stimulation

Repeats of a pure tone were used as auditory stimuli. The pure tone was 800 Hz in frequency and 25 ms in duration (rise and fall, 5 ms). The auditory stimulus was created by a personal computer (Panasonic CF-RZ6, Windows XP 32 bit) equipped with a sound card (SE-200PC, Onkyo, Osaka, Japan) and presented binaurally at a sound pressure level of 75 dB using earpieces (E-A-Rtone 3A, Aero Company, Indianapolis, IN, USA). The intensity of the sound was measured with a 2-cc coupler (Electa, Tokyo) using a sound-level meter (EL-42, Rion, Tokyo) placed at the end of the tube. The auditory stimulus consisted of a train of 48 pure tones with a total duration of 1,200 ms. Participants were stimulated on both the left and right sides in separate trials. The sound was presented *via* earpieces (E-A-Rtone 3A, Aero Company, Indianapolis, IN, USA), with the sound pressure controlled by an audiometer (AA-71, Rion, Tokyo, Japan).

**Figure 1:**
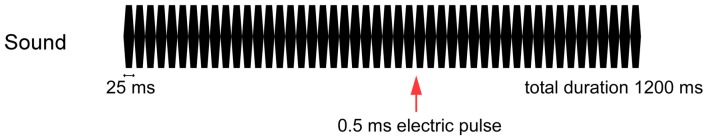
Stimulation paradigm. A 0.5-ms electrical pulse was randomly presented to the dorsum of the left or right hand at 700 ms when a train of 25-ms pure tones was applied to the left or right side at 75 dB for 1,200 ms.

The tactile stimulus used was a current-constant square wave pulse of 0.5 ms delivered to the dorsum of the left or right hand between the first and second metacarpal bones using a felt-tip bipolar electrode. The intensity of the stimulus was fixed at 2.5 times the sensory threshold. The tactile stimulus was presented both ipsilateral and contralateral to the side of sound presentation 700 ms after the onset of the sound train (Figure 1).

### MEG Recordings

Magnetic signals were recorded using a 306-channel whole-head MEG system (Vector-view, ELEKTA Neuromag, Helsinki, Finland) consisting of 102 identical triple sensor elements. Each sensor element comprised of two orthogonal planar gradiometers and one magnetometer coupled with a multi-superconducting quantum interference device, which served to provide three independent measurements of the magnetic fields. We analyzed the MEG signals recorded from 204 planar-type gradiometers, which were sufficiently powerful to detect the largest signal only over local cerebral sources. Signals were recorded with a bandpass filter of 0.1–300 Hz and were digitized at 4,000 Hz. Epochs with MEG signals larger than 2.7 pT/cm were excluded from the averaging. The waveform was digitally filtered with a bandpass filter of 37.5–42.5 Hz.

### Procedure

All experiments were performed in a quiet and magnetically shielded room. The subjects sat in a chair and watched a silent movie on a screen placed at a distance of 1.5 m in front of them throughout the experiment. The left or right auditory stimulation was randomly presented. For auditory stimulation of a given side, there were three tactile conditions (left, right and absent), thus making a total of six conditions. The six conditions were randomly presented with an even probability with a trial–trial interval of 1,500 ms. The analysis window was 100 ms before to 1,200 ms after the onset of auditory stimulation. A total of at least 100 artifact-free epochs were averaged for each condition.

### Analysis

The 40-Hz ASSRs were analyzed using the source strength waveform of the auditory cortex. Dipole analyses were performed using the Brain Electrical Source Analysis software package (GmbH, Grafefling, Germany) for each subject. The MEG waveforms of the three conditions of the left auditory stimulation were first combined. Then, the equivalent current dipole for the main component of ASSR was estimated in each hemisphere in a time window of 300–700 ms from the onset of the auditory stimulus (Lt-sound model). The same procedure was then applied to the right auditory stimulation (Rt-sound model). Next, in order to remove any somatosensory evoked cortical responses, the dipoles for the somatosensory response were included in the dipole model. MEG waveforms for the two left tactile conditions (left sound + left hand and right sound + left hand) were averaged, and the dipole in the somatosensory cortex on the right side was obtained in a time window of 700–900 ms from the onset of the auditory stimulus (Lt-tactile model). The same procedure was performed for the tactile stimulation on the right (Rt-tactile model). Once the dipoles for the somatosensory cortex and bilateral auditory cortex were established, we applied dipole models to the MEG waveforms according to the stimulus combination: the Lt-sound or Rt-sound model for the two auditory alone conditions and the Sound model + Tactile model for the four auditory and tactile conditions.

The goodness of fit (GOF) of all participants by the Sound model was over 60% (73.6 ± 8.4 and 75.3 ± 7.0% on the average for left and right auditory stimulation, respectively). Furthermore, we checked whether the presence of the tactile dipole affected the fit of the auditory dipoles in each subject (Inui et al., 2004). When the Tactile model was added to the Sound model using data for the sound alone conditions, the GOF value was improved by 0.42% ± 0.27% and 0.37% ± 0.25% for the left and right auditory stimulations, respectively. However, the difference was not statistically significant for any participant (*p* > 0.39; Inui et al., 2004), suggesting that the presence of the tactile dipole did not affect the fit of the auditory dipoles.

By using the source strength waveform, the peak of each 40-Hz wave could be measured. We defined the peak of the upward wave (anterior directing intracellular current) between 675 and 700 ms as the latency at “700 ms” and measured the peak latencies of ASSR at 25 ms intervals. Measured latencies were subtracted from each latency point (e.g., the peak latency between 675 and 700 ms was subtracted by 700 ms), and the baseline was then adjusted by the average of the latencies at 625, 650, 675 and 700 ms as a previous study (Sugiyama et al., 2019). The peak latencies of the ASSRs were compared across conditions by using two-way repeated measure ANOVA, with tactile stimulation and hemisphere as independent variables. To compare the differences between the conditions, *post hoc* multiple comparisons were performed using Bonferroni adjusted *t*-tests. All statistical analyses were performed with the level of significance set at 0.05.

## Results

**Figure 2:**
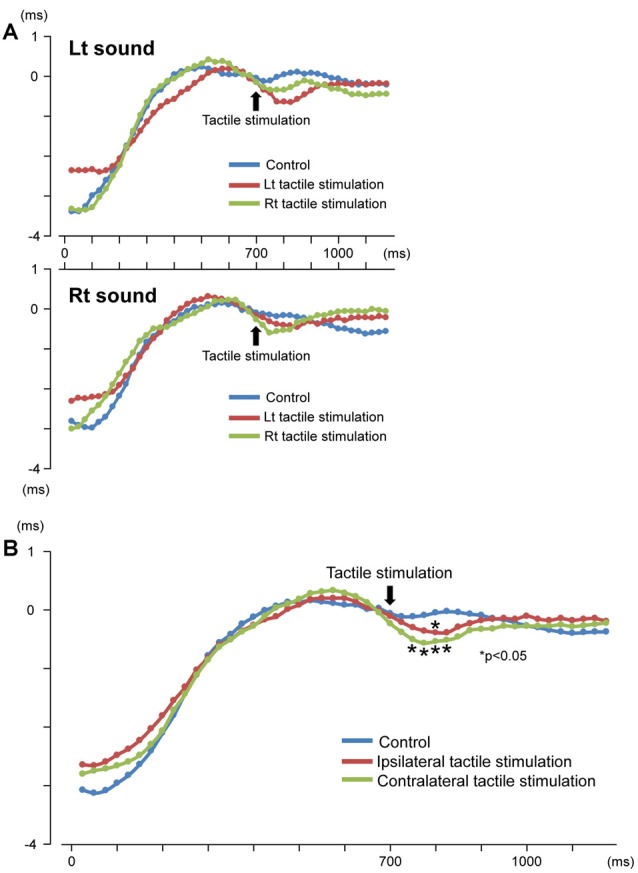
Time course of the peak latency of 40-Hz auditory steady-state response (ASSR). Mean peak latency of each ASSR sine wave is plotted for each time point. Results for the left and right sound conditions **(A)** and three conditions (control, ipsilateral and contralateral tactile stimulations to the response hemisphere) **(B)** are shown.

**Figure 3:**
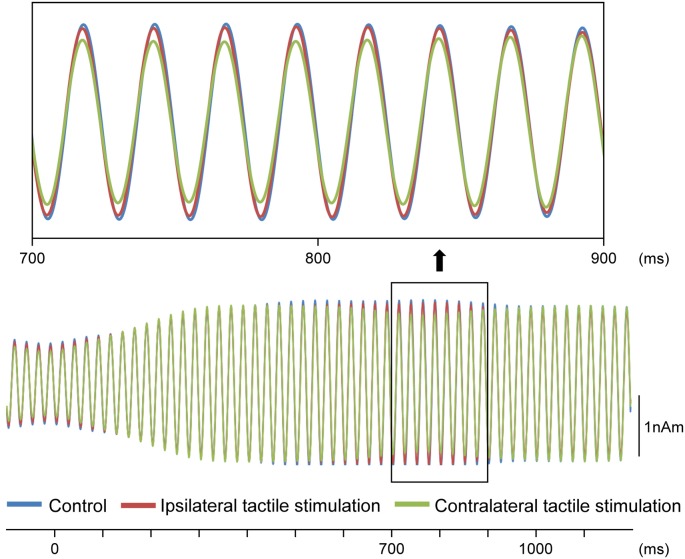
Effects of the tactile input on ASSR. Grand-averaged waveforms across 12 participants are shown.

Our results demonstrated that repeats of a pure tone elicited clear sine waves. Figure 2A shows the mean peak latency of each ASSR sine wave concerning the left and right sound conditions. Tactile stimulation served to shift the ASSR phase with the same tendency regardless of the left and right sounds. Therefore, we used two-way ANOVA without distinguishing left and right sounds. Although it was known that the ASSR amplitude showed the right hemisphere dominance (Ross et al., 2005b), ANOVA results revealed that brain hemisphere did not play a role in determining the peak latency of the ASSR at any latency point (*p* > 0.22). On the other hand, tactile stimulation significantly affected this process at 725–825 ms (*F*_(2,46)_ = 3.96–5.03; *p* = 0.011–0.026). Figure 3 shows the grand-averaged waveforms of the ASSRs combining both hemispheres. *Post hoc* testing revealed that, as compared with control conditions, contralateral tactile stimulation significantly shortened the ASSR latency at 750–825 ms (*p* = 0.014–0.028), while ipsilateral tactile stimulation was significant at 800 ms (*p* = 0.049; Figure 2B). No differences were observed between ipsilateral and contralateral tactile stimulations at any latencies after 700 ms (*p* > 0.61). On average, the peak latency of the ASSR with tactile stimulation was shorter than that without 0.41 ms at 800 ms.

## Discussion

In this study, we examined the cross-modal effects of tactile inputs on ASSR. Our results revealed that tactile stimulation clearly caused phase resetting, which was observed as a decrease in subsequent ASSR latency. ASSRs were found to be modulated by sound onset, with the latency shift approximately 3 ms (Figure 2). These results are comparable with previous reports (Ross et al., 2002, 2005a), with the effect of the phase shift from the sound change reported to be the same or slightly smaller (Ross and Pantev, 2004; Ross et al., 2005a; Sugiyama et al., 2019). Although the latency shift of the observed ASSRs by tactile input was considered a small effect (approximately 0.4 ms) compared with that by changes in auditory information itself, this study showed, for the first time, that another sensory system significantly affected the ASSR. Moreover, this finding is consistent with the notion that cross-modal interactions shorten the physiological reactions to sensory stimuli (Rowland et al., 2007).

Tactile stimulation significantly shortened ASSR latency at the sampling point of 750 ms, where the peak latency of the grand average waveform was approximately 743 ms. Therefore, somatosensory information that converged on the auditory system may have arisen during the early processing stages. We previously showed that somatosensory information takes 14.4, 18.0 and 22.4 ms from transcutaneous electrical stimulation of the dorsum of the hand to reach Brodmann’s areas 3b, 1 and 5, respectively (Inui et al., 2004). Considering that only 20–30 ms would be left to reach these auditory areas after being projected to the primary somatosensory cortex, we consider that the early stages of the somatosensory cortex, rather than the multimodal areas, represent the origin sites. Cross-modal interactions in the superior colliculus generally depend on the functional inputs from multimodal association areas (Jiang et al., 2001), thus it is also an unlikely candidate as the origin site.

Our findings regarding multimodal interactions during the early stages of sensory processing are consistent with our previous study that demonstrated that the sound presented 50 ms before tactile stimulation significantly shortened the latency of N20 m originating from Brodmann’s area 3b (Sugiyama et al., 2018). In addition, a study investigating the time course of multisensory interactions between simultaneous auditory and somatosensory stimulations also found a significant interaction in the evoked potentials at an onset latency of 50 ms (Foxe et al., 2000). While the feedback pathway from associated areas for cross-modal interactions has been described (Jiang et al., 2001, 2002, 2007; Macaluso and Driver, 2001; Schroeder and Foxe, 2002; Rowland et al., 2014), our results suggest that there is at least one interaction mechanism that does not require a feedback pathway. Indeed, our findings indicate that various stages of sensory processing in one sensory modality receive nonspecific inputs from other modalities (Ghazanfar and Schroeder, 2006; Driver and Noesselt, 2008). This notion is supported by another study in monkeys using current source density (Schroeder et al., 2001). Other studies using monkeys revealed that corticocortical information transfer follows a feedforward-type laminar organization of multimodal connections between low-level sensory areas (Cappe and Barone, 2005). Therefore, direct corticocortical or thalamocortical projections to the auditory area from the early stages of the somatosensory pathway appear to be a likely candidate for the observed multisensory interactions (Henschke et al., 2015).

In the current study, contralateral tactile stimulation significantly shortened ASSR latency at 750–825 ms (Figure 2B). Our previous study on multisensory interaction of auditory inputs to somatosensory cortex revealed that sound significantly affected N20 m latency in the range of 100 ms (Sugiyama et al., 2018), with the time course of the multimodal effect approximately consistent. Our current findings are also consistent with previous studies in that the temporal window of the observed multimodal integration is approximately within 200 ms (Giard and Peronnet, 1999; Molholm et al., 2002, 2006; Senkowski et al., 2006). Due to its reliability for measuring peak latency every 25 ms, 40-Hz ASSR is considered superior for providing information on processing speed. Indeed, our present ASSR results clearly provide evidence of accelerated sensory processing when a cross-modal event occurs.

Our findings indicate that while the reduction of ASSR latency is small compared to previously reported phase shifts, it can be considered a reliable marker of cross-modal acceleration. Ross et al. (2005a) mentioned that the cross-modal reset of ASSR if it existed, was suspected to be much smaller than the within modality reset. This suggestion was consistent with our results. However, the shortened time of roughly 0.4 ms observed in this study was much smaller than that reported by psychophysical studies; the reaction time to audio-tactile stimulation is approximately 25 ms shorter than that to unimodal stimulation (Murray et al., 2005). Although how brain areas contribute to the acceleration of the ultimate motor reaction remains unclear, it is conceivable that the sensory, multimodal, and motor areas contribute by both augmenting and quickening the responses. If there are multisensory interactions at each stage of the hierarchical sensory processing and motor execution, then the reduction in the final response time must reflect the cumulative effects.

There are some limitations in the present study. Participants in this study watched a silent movie to reduce their burden. The second purpose was to minimize attention effects (Ross et al., 2004) by concentrating on a silent movie and ignoring sensory stimuli. Since the visual stimulus derived from a silent movie was not time-locked to auditory stimuli, it is unlikely that the visual-auditory interaction played an important role in the present results. Nevertheless, effects on the ASSR latency could not be completely denied. Another limitation is sex differences between subjects. We could only recruit three women out of 12 subjects. Therefore, we could not analyze sex differences (Melynyte et al., 2018). Finally, we focused on the ASSR latency rather than the amplitude in this study. Therefore, the ASSR latency might have been affected by the reduction of the ASSR amplitude.

## Conclusions

Rapid processing of sensory information is necessary for animals to survive and is considered a basic objective of multisensory interactions. To our knowledge, the present study is the first to report that tactile cross-modal interactions cause phase resetting, with a resulting reduction in ASSR latency. This reduced ASSR latency indicated that a new sensory event by cross-modal inputs increased the speed of ongoing sensory processing.

## Data Availability Statement

All datasets generated for this study are included in the article.

## Ethics Statement

The studies involving human participants were reviewed and approved by Ethics Committee of the National Institute for Physiological Sciences, Okazaki, Japan. The patients/participants provided their written informed consent to participate in this study.

## Author Contributions

SS and KI designed the work. SS, TK, NT, MN and KI performed the experiments. SS analyzed the data. SS and KI drafted the manuscript. TS commented. All authors read and approved the manuscript.

## Conflict of Interest

The authors declare that the research was conducted in the absence of any commercial or financial relationships that could be construed as a potential conflict of interest.
